# An angiogenesis-related lncRNA signature predicts the immune microenvironment and prognosis of breast cancer

**DOI:** 10.18632/aging.204930

**Published:** 2023-08-03

**Authors:** Ya-Wen Wang, Can Liu, Yan-Duo Chen, Bin Yang, Xu Chen, Guangxin Ma, Ya-Ru Tian, Xiangkun Bo, Kai Zhang

**Affiliations:** 1 Department of Breast Surgery, General Surgery, Qilu Hospital of Shandong University, Jinan, People’s Republic of China; 2 Department of Pathology, Qilu Hospital of Shandong University, Jinan, People’s Republic of China; 3 Hematology and Oncology Unit, Department of Geriatrics, Qilu Hospital of Shandong University, Jinan, People’s Republic of China; 4 Department of Radiation Oncology, Shandong Cancer Hospital and Institute, Shandong First Medical University and Shandong Academy of Medical Science, Jinan, People’s Republic of China; 5 Department of General Surgery, Affiliated Haian Hospital of Nantong University, Nantong, People’s Republic of China

**Keywords:** breast cancer, angiogenesis-related lncRNA, risk model, immune microenvironment, prognostic value

## Abstract

Both angiogenesis and lncRNAs play crucial roles in the development and progression of breast cancer. Considering the unknown association of angiogenesis and lncRNAs in breast cancer, we aim to identify angiogenesis-related lncRNAs (ARLs) and explore their prognostic value. Here, based on analysis of The Cancer Genome Atlas database, the correlation between ARL and the prognosis and immune infiltration landscape of breast cancer were investigated. Eight ARLs (MAFG−DT, AC097478.1, AL357054.4, AL118556.1, SNHG10, MED14OS, OTUD6B−AS1, and CYTOR) were selected to construct the risk model as a prognostic signature. The survival rate of the patients in the high-risk group was lower than that in the low-risk group. The ARL signature was an independent prognostic predictor, and areas under the curve of 1-, 3-, and 5-year survival were 0.745, 0.695, and 0.699, respectively. The prognostic ARLs were associated with the immune infiltration landscape and could indicate the immune status, immune response, tumor mutational burden, and drug sensitivity of patients with breast cancer. Furthermore, qRT-PCR of clinical samples revealed that OTUD6B−AS1 was correlated with prognostic pathological parameters. OTUD6B−AS1 promoted breast cancer cell proliferation, wound healing, migration, invasion, and human umbilical vein endothelial cells tube formation. Mechanistically, OTUD6B−AS1 regulated EMT- and angiogenesis-related molecules. Taken together, we constructed and verified a robust signature of eight ARLs for the prediction of survival in patients with breast cancer, and the characterization of the immune infiltration landscape. Our findings suggest that OTUD6B−AS1 could be a therapeutic target for patients with breast cancer.

## INTRODUCTION

According to the data on the global burden of cancer, breast cancer has emerged as the prevailing form of malignancy [1]. Breast cancer ranks among the most perilous forms of cancer in females [2]. The prognosis for patients with breast cancer has greatly improved due to the implementation and advancement of diverse treatment approaches, including chemotherapy, radiation therapy, immunotherapy, and hormonal therapy. However, many patients with breast cancer still suffer relapse, metastasis, and resistance to therapy [3, 4]. Therefore, the search for efficient early indicators or targets with possible clinical application will have a vital impact on the patients’ outcomes.

Angiogenesis is necessary for the growth and metastasis of invasive tumors [5]. Development and metastasis of breast cancer heavily rely on angiogenesis, which is not initiated during the early stages of tumor formation [6]. The angiogenesis state enables tumors to recruit new capillaries, which in turn supply oxygen and nutrients to neighboring cells, leading to rapid tumor growth [7]. In breast cancer, the level of angiogenesis is one of the prognostic markers [8]. Increased levels of growth factors involved in angiogenic process are associated with breast cancer aggressiveness [9, 10]. Furthermore, presence of microvessels in invasive breast cancer could potentially act as an indicator for metastasis or recurrence [11]. Hence, it holds immense importance to further reveal the mechanism and clinical possibilities of angiogenesis in breast cancer.

LncRNAs (long noncoding RNAs) participate in many biological processes by regulating gene expressions, via transcriptional regulation, posttranscriptional regulation and epigenetic regulation of chromatin modification [12]. LncRNAs are involved in breast cancer carcinogenesis by regulating cell proliferation, invasion, migration, apoptosis, drug resistance and epithelial–mesenchymal transformation (EMT) [13]. Extensive research and clinical application of lncRNAs in breast cancer have revealed that numerous lncRNAs show promise as biomarkers and targets, highlighting their significant potential [13]. Recently, the impact of lncRNA on tumor angiogenesis has drawn increasing attention [14]. It has been reported that lncRNAs affect angiogenesis in tumor development through different ways, including competing endogenous RNAs, signaling pathway regulation, expression-level regulation of angiogenic factors and their receptors, recruitment of RNA polymerase, and gene transcription [15]. Certainly, the lncRNA SNHG1 has demonstrated its role as a regulator of M2 macrophage polarization by inhibiting STAT6 phosphorylation and controlling the growth of tumors and angiogenesis in breast cancer [16]. However, the impact of ARL (angiogenesis-related lncRNA) signature on prognosis of breast cancer and immune microenvironment is not yet clearly clarified.

Here, based on The Cancer Genome Atlas (TCGA) database, the correlation between ARL and prognosis of breast cancer was investigated. Afterwards, a risk model was developed based on eight ARLs to predict the prognosis of patients with breast cancer. The correlation of the risk score with immune infiltration landscape was analyzed. Potential molecular signaling pathways were also predicted. Exploration was conducted on the expression of OTUD6B–AS1 in breast cancer tissues, as well as its impact on cellular behaviors and regulation of signaling pathways associated with EMT and angiogenesis.

## RESULTS

### Identification of prognostic ARLs

**Figure 1:**
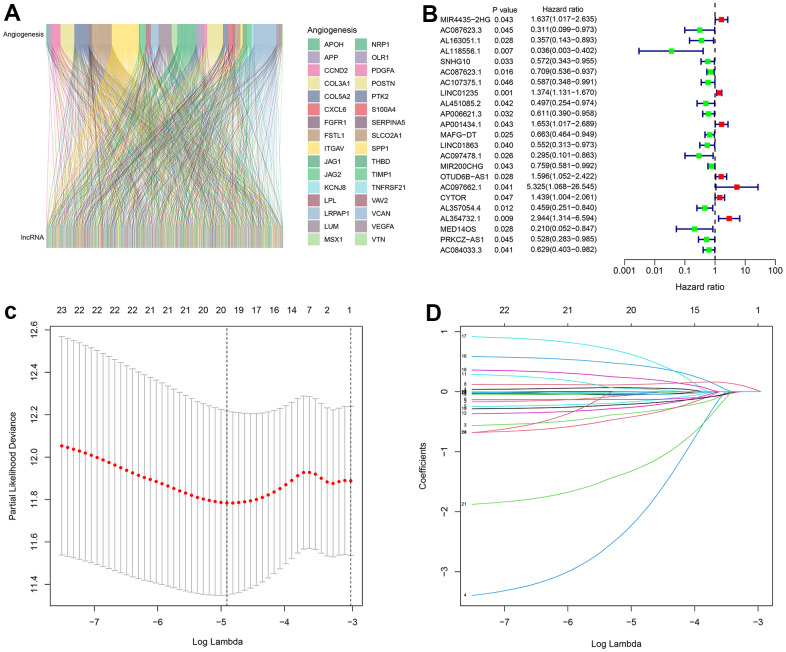
**Identification of prognostic ARLs in breast cancer.** (**A**) Sankey diagram showing the correlation between angiogenesis genes and lncRNAs. (**B**) Univariate Cox regression analyses suggest that 23 ARLs were significantly correlated with the overall survival (OS) of patients with breast cancer. (**C**, **D**) LASSO analyses showing the minimum lambda and coefficient of the prognostic ARLs.

A grand total of 14,142 long non-coding RNAs were discovered from the breast cancer RNA-Seq matrix in FPKM format. Based on the “limma” script, the RNA-Seq matrix of each breast cancer sample from the TCGA database was transformed from FPKM into TPM. To screen lncRNAs related to angiogenesis, 36 angiogenesis genes were found and a Pearson correlation was conducted to screen the ARLs. In the end, a total of 464 lncRNAs linked to the process of angiogenesis were discovered (Figure 1A and Supplementary Table 1). Figure 1B demonstrated that there were 23 ARLs associated with overall survival (OS) through the analysis of univariate Cox regression. Then according to LASSO analysis, 20 prognostic ARLs were chosen for further analysis (Figure 1C, 1D).

### Developing a risk model utilizing an ARL prognostic signature

**Figure 2:**
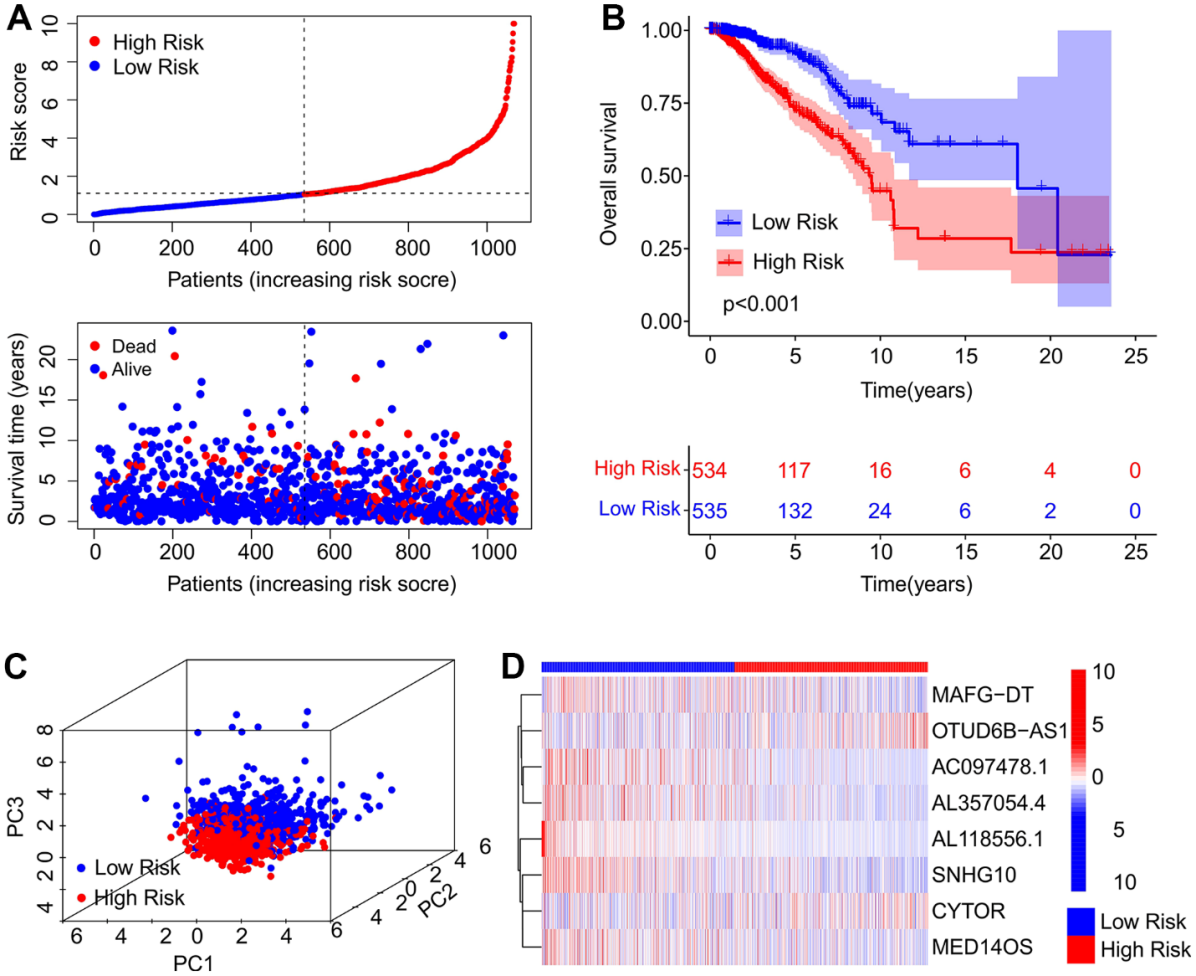
**Risk model construction of the prognostic ARLs.** (**A**) Distribution of risk score and scatter dot plot showing the correlation of survival time and risk score. (**B**) Kaplan-Meier survival curve suggests that the OS of patients in the high-risk group is significantly shorter than that in the low-risk group. (**C**) Principal component analysis (PCA) illustrates a significant difference between the low-risk group and high-risk group based on the ARL prognostic signature. (**D**) Heatmap showing the expression of the eight prognostic ARLs in the low- and high-risk groups.

A new risk model was developed to assess the significance of the ARL signature in patients with breast cancer. The prognostic risk model was established by selecting eight ARLs through multivariate Cox regression. Based on the median value of risk scores, the patients were classified into high-risk group and low-risk group. The scatter plot demonstrated an inverse relationship between risk scores and survival time (Figure 2A). Overall survival (OS), progress free interval (PFI) and disease specific survival (DSS) time of patients with high-risk scores were shorter than low-risk score patients (P < 0.001, Figure 2B and Supplementary Figure 1). Principal component analysis (PCA) showed a separation between high-risk group and low-risk group (Figure 2C). Heatmap visualization results demonstrated a significant different expression level of eight ARLs between the two groups. The low-risk group revealed higher expression of MAFG–DT, AC097478.1, AL357054.4, AL118556.1, SNHG10, and MED14OS, but lower expression of OTUD6B–AS1 and CYTOR (Figure 2D).

### Performance of the risk model in the training and validation cohorts

**Figure 3:**
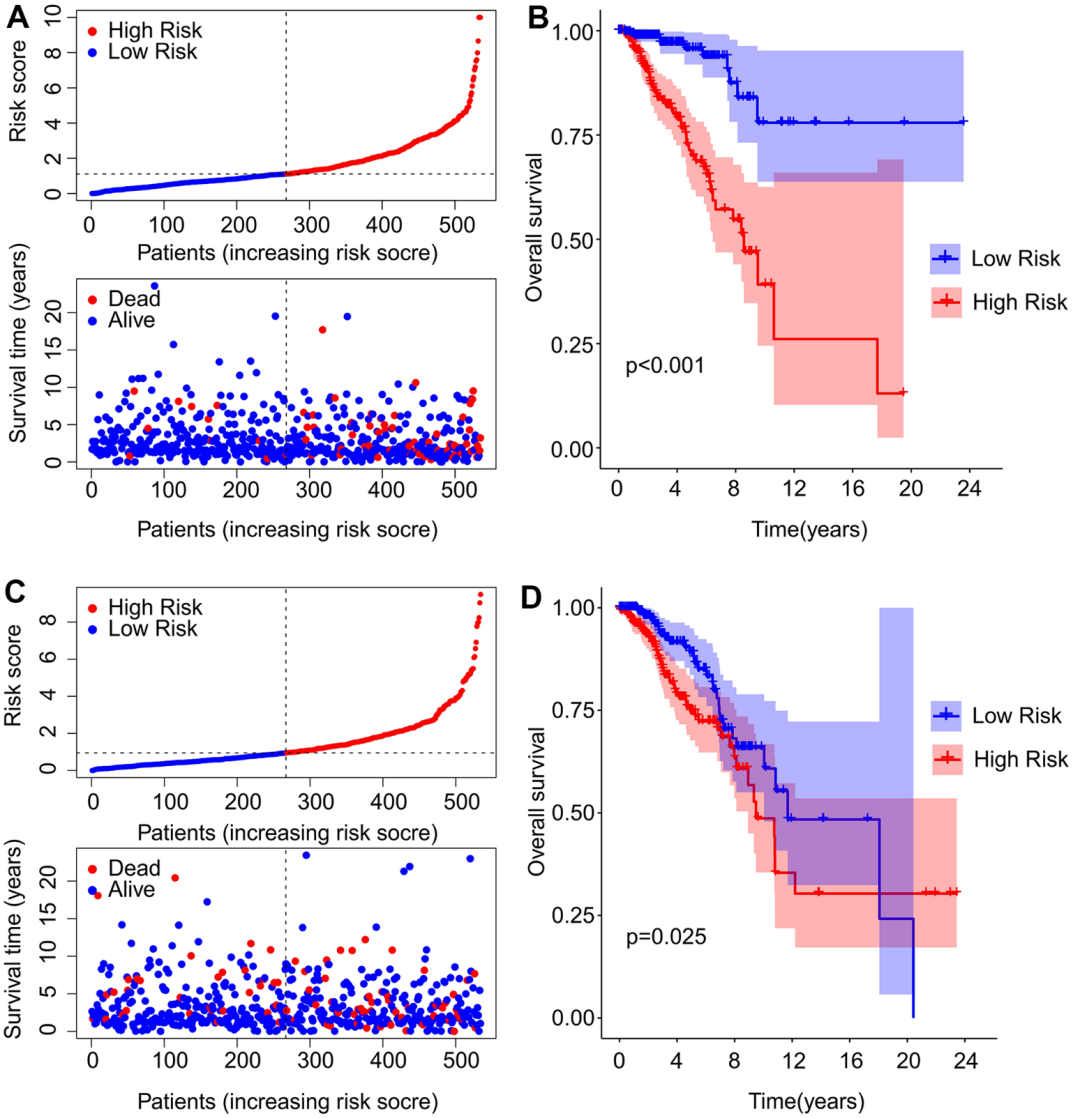
**Construction of the risk model in the training and validation cohorts.** (**A**) The distribution of risk score and scatter dot plot shows the correlation of risk score and OS in the training cohort. (**B**) Kaplan-Meier survival curve suggests that the OS of patients with high-risk score is significantly lower than those with low-risk score in the training cohort. (**C**) The distribution of risk scores and scatter dot plot of patients with breast cancer based on the ARL prognostic signature in the validation cohort. (**D**) Kaplan-Meier survival curve suggests that the OS of patients with high-risk score is significantly lower than those with low-risk score in the validation cohort.

To confirm the efficacy of the model, breast cancer patients were divided into training and validation cohorts at a ratio of 7 to 3. In the training cohort, the scatter diagram indicated a negative correlation between the patients’ survival time and the risk score (Figure 3A). Kaplan-Meier analysis indicated that patients with high risk scores had a significantly lower overall survival rate compared to those with low risk scores (P < 0.001, Figure 3B). Similar trends were observed in the validation cohort as evidenced by scatter plot and Kaplan-Meier analysis (Figure 3C, 3D). The OS rate in high-risk group was notably lower compared to that in low-risk group (P = 0.025, Figure 3D).

### The ARL prognostic signature as an independent prognostic factor

**Figure 4:**
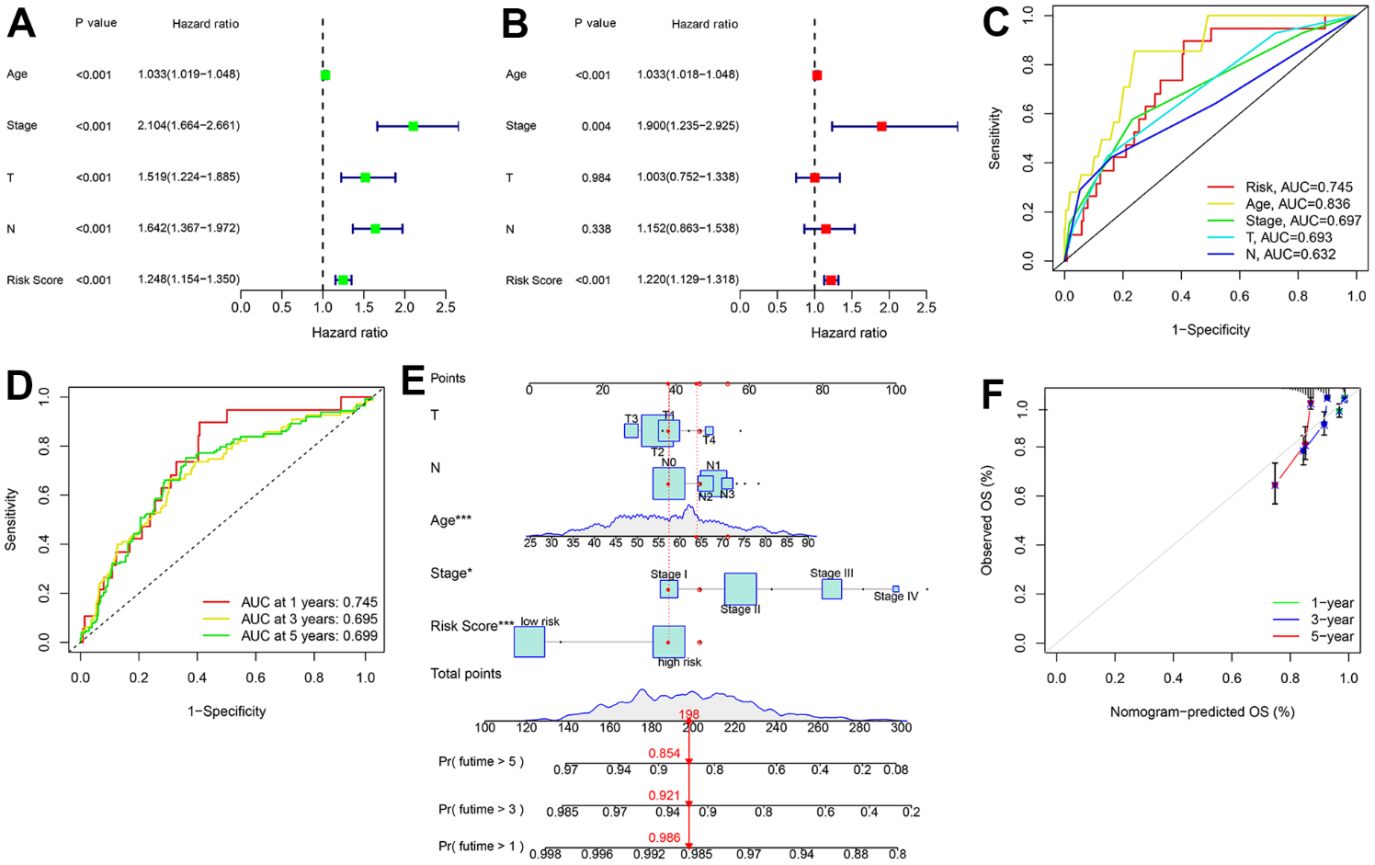
**Independent prognostic analysis of ARL.** (**A**) Univariate Cox regression analysis shows the correlation between OS rate and clinicopathological parameters, including age, stage, T stage, N stage, and the ARL prognostic signature risk score. (**B**) Multivariate Cox regression analysis shows that age, stage, and risk score are independent prognostic predictors of patients with breast cancer. (**C**) Receiver operating characteristic curve (ROC) shows the areas under the curve (AUC) of the prognostic signature and clinical characteristics. (**D**, **E**) Nomogram construction of the prognostic signature and clinicopathological parameters to predict the 1-, 3- and 5-year survival rates of patients with breast cancer. Time-dependent ROC curve shows the 1-, 3-, and 5-year AUC. (**F**) Calibration curve reveals the accuracy between predictive power and actual survival of 1-, 3-, and 5-year survival.

Cox regression analyses were performed to investigate whether the ARL prognostic model is an independent prognostic predictor of breast cancer. Univariate analysis showed that Age (hazard ratio [HR] = 1.033, P < 0.001), stage (HR = 2.104, P < 0.001), stage T (HR = 1.519, P < 0.001), stage N (HR = 1.642, P < 0.001) and risk score (HR = 1.248, P < 0.001) were significantly related with OS of breast cancer (Figure 4A). Multivariate analysis showed that age (HR = 1.033, P < 0.001), stage (HR = 1.900, P = 0.004), and risk score (HR = 1.220, P < 0.001) were independent prognostic predictors (Figure 4B). The ROC curve results showed that the AUC for risk score was 0.745 (Figure 4C).

Additionally, we constructed a new nomogram model integrating risk score and clinicopathological features (Figure 4D, 4E). The consistency index (C-index) of the nomogram was 0.737. The ROC curve showed AUC of 0.745, 0.695, and 0.699 for 1-year, 3-year, and 5-year survival, respectively (Figure 4D, 4E). Calibration curves demonstrated that the survival rates predicted by the nomogram were consistent with actual survival time of breast cancer patients (Figure 4F).

### Subgroup analysis of the ARL risk model based on clinicopathological characteristics

**Figure 5:**
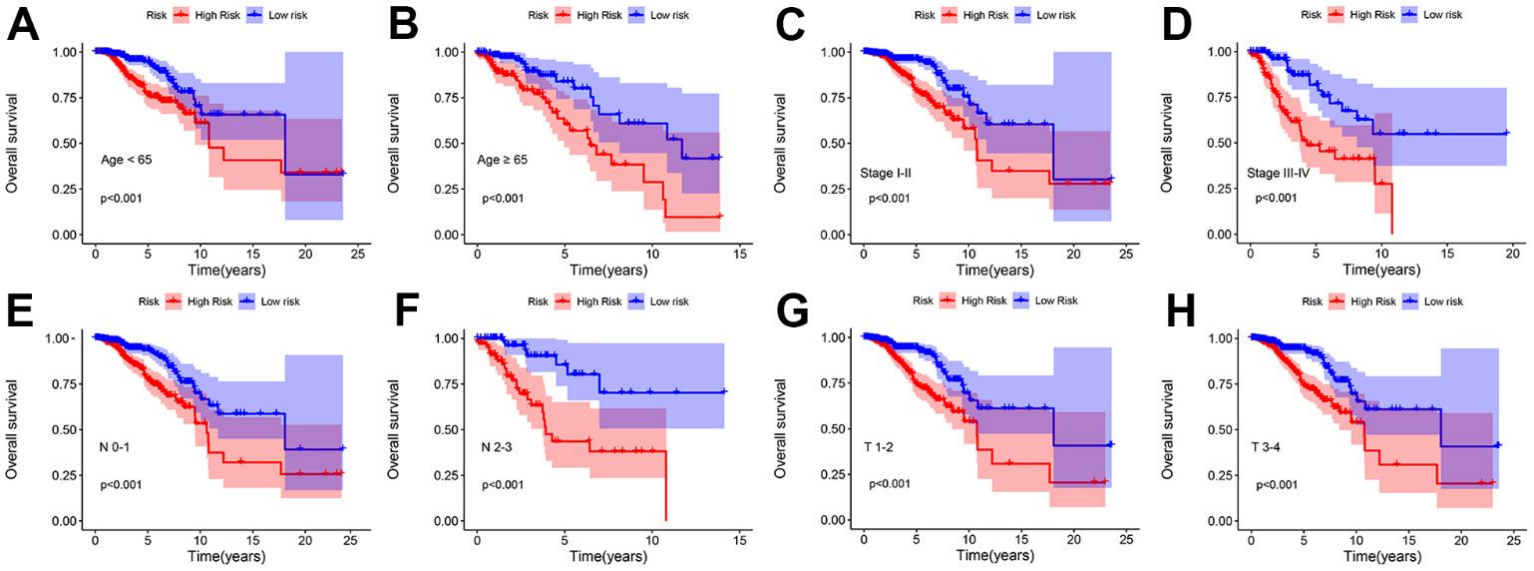
**Kaplan–Meier survival curve of patients with breast cancer stratified by different clinicopathological characteristics.** Kaplan**–**Meier survival curve analysis shows the OS rates of low- and high-risk patients with breast cancer stratified by (**A**, **B**) age < 65 vs. ≥ 65, (**C**, **D**) Stage I–II vs. Stage III–IV, (**E**, **F**) N 0–1 vs. N 2–3, (**G**, **H**) T I–II vs. T III–IV.

The patients were categorized into different subgroups according to age (< 65 vs. ≥ 65 years), N stage (N 0–1 and N 2–3), stage (stage I–II and stage III–IV), and T stage (T I–II and T III–IV). The Kaplan–Meier survival curve indicated that the high-risk patients group had a decreased overall survival rate in all the subgroups (Figure 5A–5H).

### Analysis of the landscape of immune cell infiltration (ICI) and evaluation of immune response

**Figure 6:**
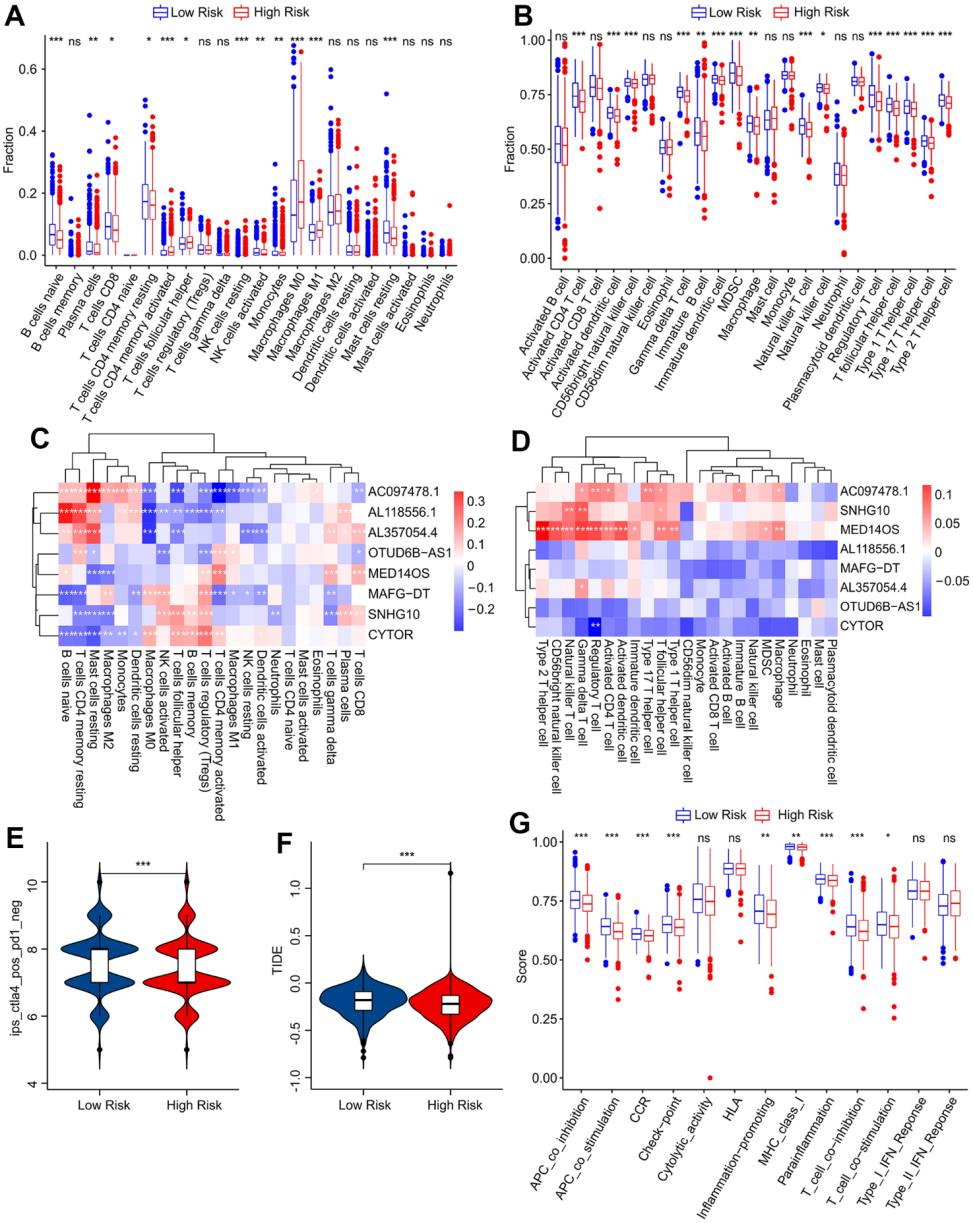
**Immune infiltration landscape and immune response analysis of patients with breast cancer.** The fraction of immune cells calculated by (**A**) CIBERSORT and (**B**) ssGSEA algorithms. (**C**) Correlation analysis of prognostic ARL and 22 types of immune cells. (**D**) Correlation analysis of prognostic ARL and 23 types of immune cells. (**E**) Immunophenoscore (IPS) analysis. (**F**) Tumor immune dysfunction and exclusion (TIDE) analysis. (**G**) Immune function score of patients in low-risk group and high-risk group.

The CIBERSORT algorithm was used to calculate the proportions of 22 immune cells based on risk stratification (Figure 6A) and it was shown that the fractions of naïve B cells, plasma cells, CD8+ T cells, CD4+ resting memory T cells, activated NK cells, resting mast cells, and monocytes were significantly higher in the low-risk group, whereas CD4 activated memory T cells, resting NK cells, follicular helper T cells, M0 and M1 macrophages were lower in the low-risk group. According to the ssGSEA algorithm, the low-risk group exhibited significantly higher proportions of immune cells (Figure 6B). To explore possible connections between predictive ARLs and immune cells, Pearson correlation analyses were employed. As displayed in Figure 6C, a clear correlation was observed between the eight prognostic ARLs and 22 immune cells, as the CIBERSORT algorithm determined. By the ssGSEA algorithm, we found that AC097478.1, SNHG10, and MED14OS were positively correlated with most immune cell subtypes (Figure 6D). Patients with breast cancer were further evaluated for their responses to anti-CTLA-4 immunotherapy and anti-PD-1 immunotherapy, taking into account the variations in the ICI landscape based on risk stratification. Promising response to anti-CTLA-4 was observed in the low-risk category based on the immunophenoscore (IPS) findings (Figure 6E). Furthermore, TIDE analysis demonstrated that patients with breast carcinoma in the high-risk category exhibited a more favorable reaction to immunotherapeutic treatment (Figure 6F). Immune function analysis revealed low-risk patients were associated with higher immune scores in several terms, such as antigen-presenting cell (APC) and CC chemokine receptor (CCR) (Figure 6G).

### Tumor mutational burden (TMB) analysis

**Figure 7:**
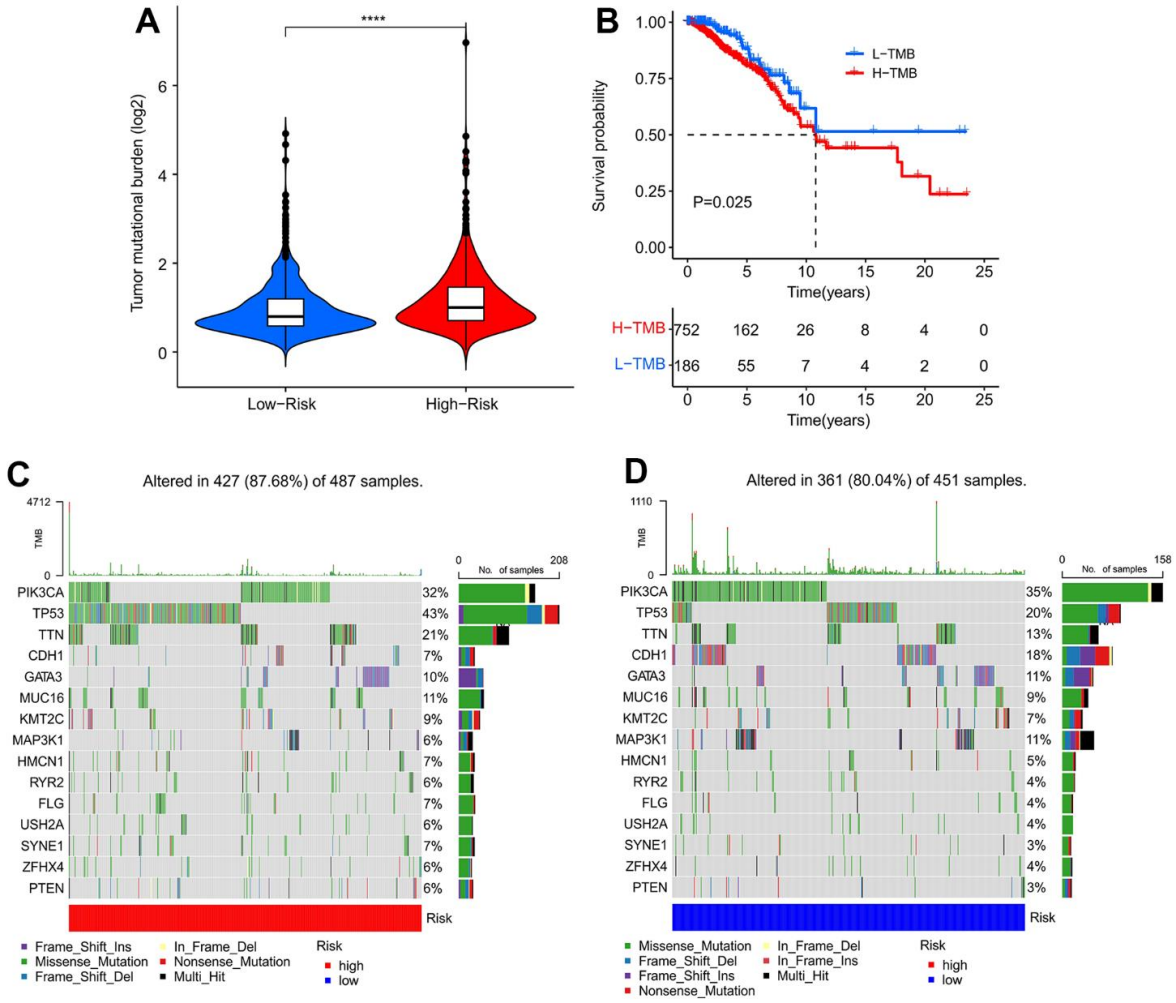
**Tumor mutational burden (TMB) analysis of patients with breast cancer.** (**A**) TMB analysis of patients in high-risk group and low-risk group. (**B**) Kaplan–Meier survival curve shows that the OS of patients with low TMB (L-TMB) is longer than that of patients with high TMB (H-TMB). (**C**, **D**) Mutational burden landscape of patients in high-risk group and low-risk group.

The TMB in high-risk patients exceeded that of the low-risk group (Figure 7A). Patients with high TMB were associated with worse OS (Figure 7B). The top 15 mutation frequency genes in both groups were further investigated, and the mutation frequencies of PIK3CA, TP53, TTN, and CDH1 in the high-risk group were 32%, 43%, 21%, and 7%, respectively (Figure 7C). Meanwhile, the mutation frequencies of PIK3CA (35%) and CDH1 (18%) were higher and those of TP53 (20%) and TTN (13%) were lower in low-risk patients (Figure 7D).

### Drug sensitivity analysis

**Figure 8:**
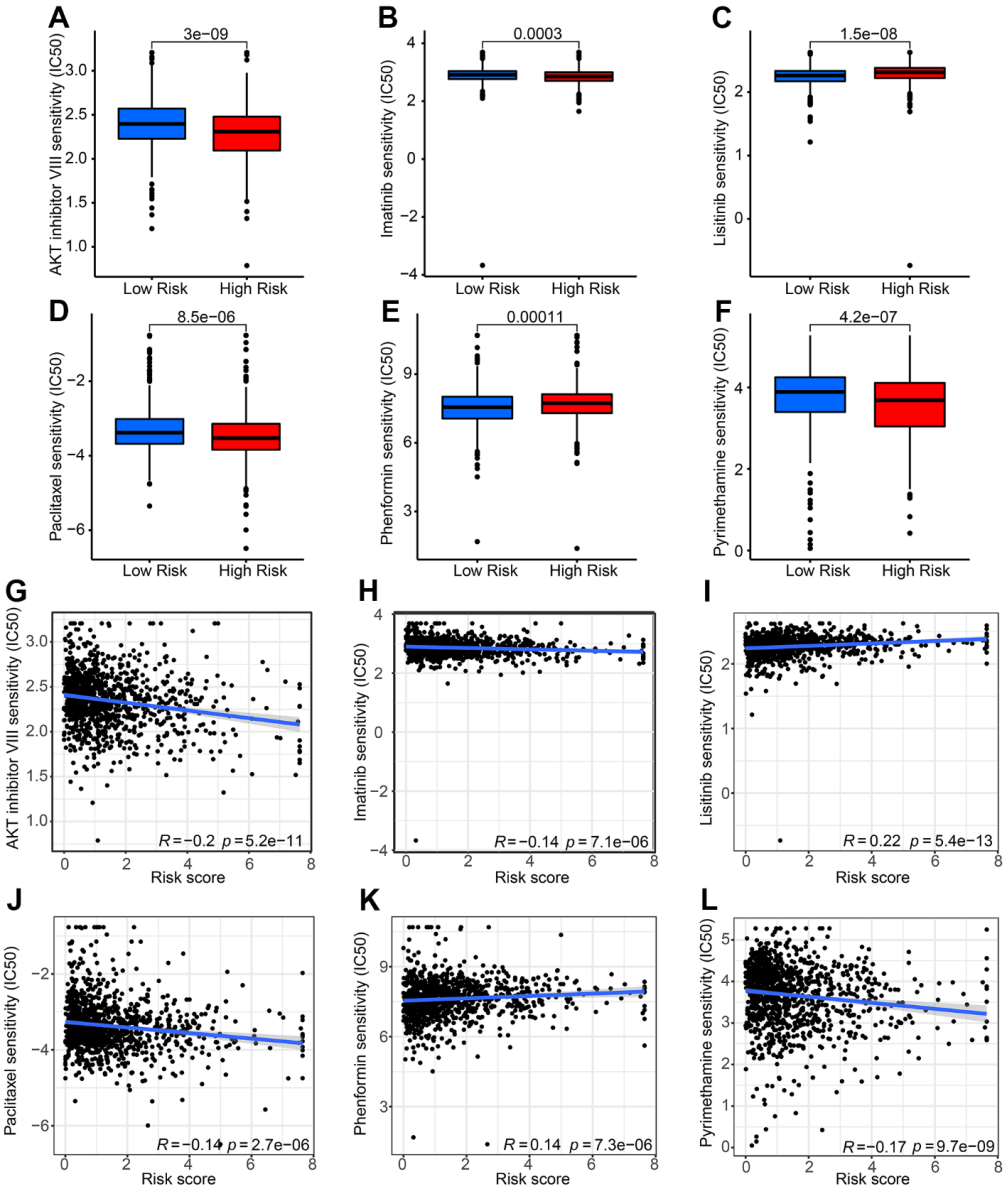
**Drug sensitivity analysis of patients with breast cancer in low-risk group and high-risk group.** The distribution of IC50 values shows a significant difference between patients in the low-group and high-risk group among (**A**) AKT inhibitor VIII, (**B**) imatinib, (**C**) linsitinib, (**D**) paclitaxel, (**E**) phenformin, and (**F**) pyrimethamine. (**G**–**L**) Correlation analysis of risk score and drug sensitivity.

The impact of risk stratification on antineoplastic drug sensitivities was further investigated. Patients with low-risk stratification exhibited higher IC50s for the AKT inhibitor VIII, imatinib, paclitaxel, and pyrimethamine. Conversely, high-risk patients displayed higher IC50s for linsitinib and phenformin (Figure 8A–8F). The risk score showed significant negative correlations with AKT inhibitor VIII (r = –0.2, P = 5.2e-11), imatinib (r = –0.14, P = 7.1e-06), paclitaxel (r = –0.14, P = 2.7e-06), and pyrimethamine (r = –0.17, P = 9.7e-09). However, it exhibited positive correlations with linsitinib (r = 0.22, P = 5.4e-13) and phenformin (r = 0.14, P = 7.3e-06) (Figure 8G–8L).

### Analysis of functional enrichment

**Figure 9:**
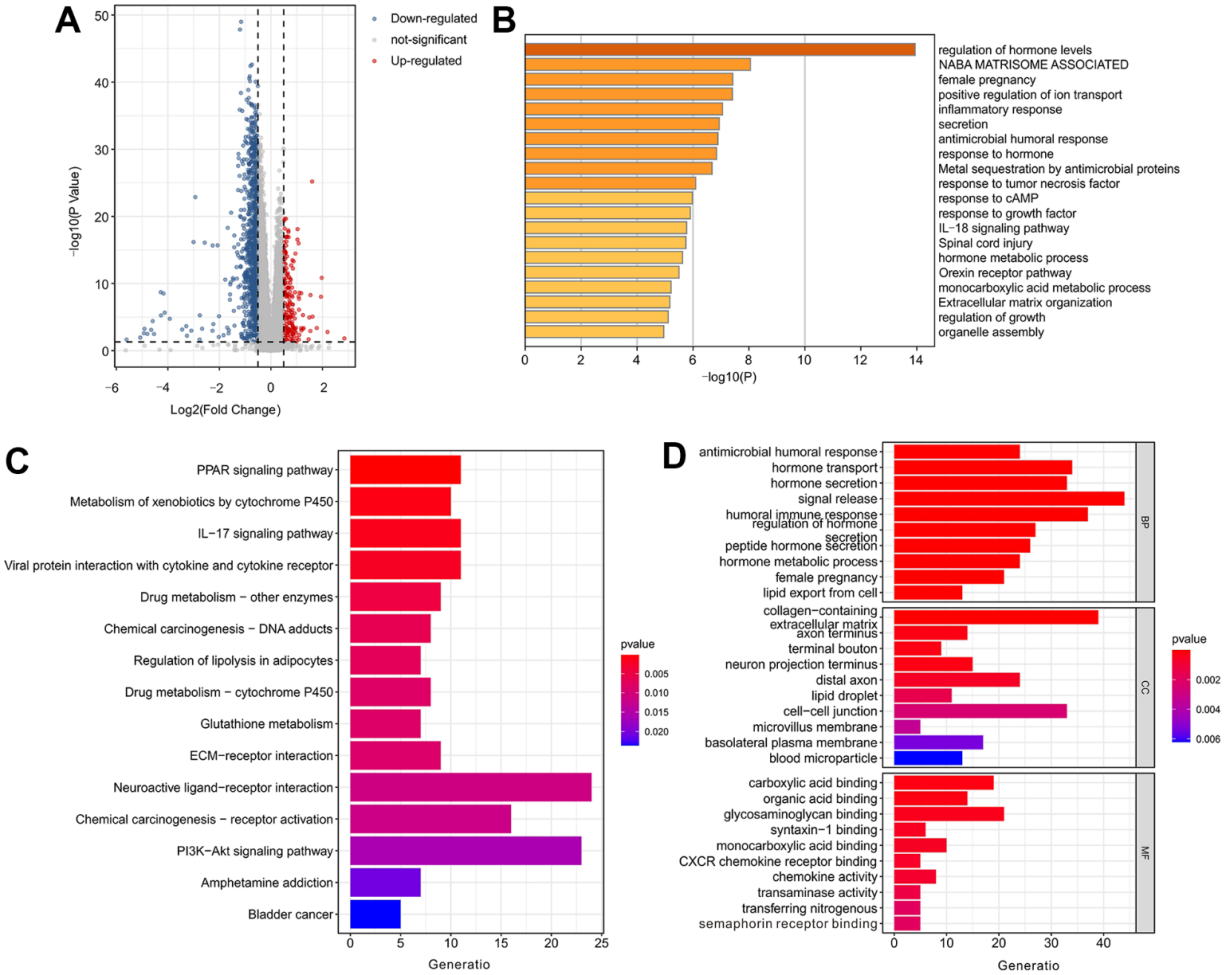
**Functional enrichment analysis of differentially expressed genes (DEGs) in low-risk group and high-risk group.** (**A**) Volcano diagram shows the DEGs in low-risk group and high-risk group with the threshold setting at |log_2_FC| ≥ 0.5 and P-value < 0.05. (**B**) Enrichment analysis of DEGs. (**C**) The top 15 KEGG enrichment analysis and (**D**) top 10 GO enrichment analysis of DEGs.

To investigate the mechanism of dysregulated genes in patients by risk stratification, functional enrichment analysis was performed. A volcano diagram showed the differentially expressed genes (DEGs) in the low-risk group and high-risk group (Figure 9A). The DEGs were enriched in the regulation of hormone levels (Figure 9B). The result of KEGG enrichment indicated that the DEGs were enriched in the PPAR pathway, metabolism of xenobiotics by cytochrome P450, IL-17 pathway and etc. (Figure 9C). According to the GO enrichment analysis, it was observed that the DEGs played a role in the antimicrobial humoral response, hormone transport, and hormone secretion (Figure 9D).

### Association of OTUD6B−AS1 with invasive pathological parameters

**Figure 10:**
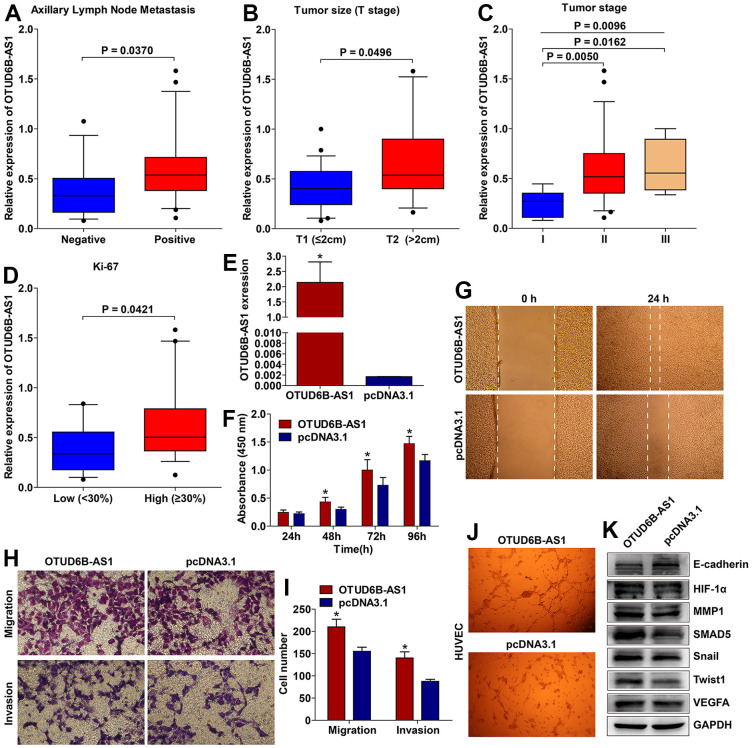
**OTUD6B−AS1 was associated with aggressive pathological parameters and promoted breast cancer progression.** Expression of OTUD6B−AS1 was positively correlated with axillary lymph node metastasis (**A**), larger tumor size (**B**), advanced tumor stage (**C**, one way ANOVA, P = 0.0096; stage I vs. II, P = 0.0050; stage I vs. III, P = 0.0162; stage II vs. III, not significant), and high Ki-67 expression (**D**). The OTUD6B−AS1 overexpression plasmid significantly upregulated OTUD6B−AS1 expression in breast cancer BT474 cells (**E**). OTUD6B−AS1 promoted breast cancer cell proliferation (**F**), wound healing (**G**, 40× magnification), migration, and invasion (**H**, **I**, 200× magnification). OTUD6B−AS1 increased the tube formation ability of HUVEC (**J**, 40× magnification). OTUD6B−AS1 decreased E-cadherin expression and increased the expression of HIF-1α, MMP1, SMAD5, Snail, Twist1, and VEGFA (**K**, cropped gels blots are used).

Among the eight prognosis-related lncRNAs, OTUD6B−AS1 was selected for further qRT–PCR validation because it showed the largest HR in the univariate Cox regression analysis and the largest coefficient in the formula of multivariate analysis. The expression level of OTUD6B−AS1 was even higher in breast cancer tissues with axillary lymph node metastasis (ALNM) than those without ALNM (Figure 10A and Table 1). Moreover, higher expression of OTUD6B−AS1 was observed in breast cancer tissues with larger tumor size (T stage) (Figure 10B), and high expression of OTUD6B−AS1 was positively related with advanced tumor stage (Figure 10C and Table 1). OTUD6B−AS1 was upregulated to a greater extent in breast cancer tissues with high (≥ 30%) Ki-67 expression compared with those with low (< 30%) Ki-67 expression [17, 18] (Figure 10D). Interestingly, OTUD6B−AS1 expression was also correlated with molecular subtypes (Table 1). These results suggested that OTUD6B−AS1 is correlated with aggressive pathological parameters, which are associated with a poor prognosis.

**Table 1:** Association between OTUD6B−AS1 expression and clinicopathological parameters in breast cancer.

**Variables**	**n**	**OTUD6B−AS1**		***P* value**
		**Low**	**High**	
**Age (y)**				
≤50	16	10	6	
>50	20	8	12	0.315
**Tumor grade**				
1	2	1	1	
2	18	10	8	
3	16	7	9	0.790
**Tumor size (T stage)**				
T1 (≤2cm)	22	13	9	
T2 (2<cmT≤5cm)	14	5	9	0.305
**Axillary lymph node metastasis**				
Negative	15	11	4	
Positive	21	7	14	**0.041**
**N stage**				
N0	15	11	4	
N1	17	6	11	
N2	3	1	2	
N3	1	0	1	0.108
**Tumor stage**				
I	8	8	0	
II	24	9	15	
III	4	1	3	**0.005**
**ER (estrogen receptor)**				
Negative	14	9	5	
Positive	22	9	13	0.305
**PR (progesterone receptor)**				
Negative	15	9	6	
Positive	21	9	12	0.500
**HER2**				
Negative	26	11	15	
Positive	10	7	3	0.264
**Ki-67**				
Low (<30%)	17	10	7	
High (≥30%)	19	8	11	0.505
**Molecular subtypes**				
Luminal A	5	4	1	
Luminal B	14	3	11	
HER2-enriched	10	7	3	
Triple-negative	7	4	3	**0.044**

### OTUD6B−AS1 promoted breast cancer progression and involved in EMT- and angiogenesis-related signaling

To investigate the function of OTUD6B−AS1, the BT474 breast cancer cell line was transfected with OTUD6B−AS1 overexpression or empty plasmids. Confirmation of transfection efficiency was achieved through qRT-PCR analysis (Figure 10E). Overexpression of OTUD6B−AS1 endowed breast cancer cells with increased proliferation (Figure 10F), wound healing (Figure 10G), and migratory and invasion (Figure 10H, 10I) abilities. HUVEC were cultured using the conditioned medium obtained from transfected BT474 cells. Subsequently, the tube formation assay was conducted. Figure 10J demonstrated that OTUD6B−AS1 enhanced the tube formation capacity of HUVEC. The expression of E-cadherin was reduced by OTUD6B−AS1, while the expressions of HIF-1α, MMP1, SMAD5, Snail, Twist1, and VEGFA were increased (Figure 10K). This indicates that OTUD6B−AS1 plays a role in controlling EMT and angiogenesis.

## DISCUSSION

Despite the considerable enhancement in the survival rate of patients with breast cancer, the current outcome is still deemed inadequate [19]. Hence, it is highly important to identify potential biomarkers for the diagnosis and treatment purposes. Here we established and validated an eight-lncRNA signature that could forecast the prognosis of patients with breast cancer. Wang et al. reported a mitochondrial function-related lncRNA signature predicting survival in breast cancer, with AUC for 1-, 3-, and 5-year survival of 0.769, 0.693, and 0.659, respectively [20]. Moreover, Yang et al. developed a lncRNA signature that predicts survival in breast cancer by considering endocrine resistance and immune factors, achieving AUC values of 0.710, 0.649, and 0.672 at 1, 3, and 5 years, respectively [21]. Chen et al. developed a predictive lncRNA signature associated with necroptosis in breast cancer, achieving AUC values of 0.731, 0.643, and 0.641 for 1, 3, and 5 years, respectively [22]. We believe that the performance of the current model is comparable or even better than the existing signatures. However, we could not exclude the possibility that the difference between the current model and existing signatures may be due to different study cohorts and/or algorithms. Comparison of the model with existing signatures in the same cohort may better address this issue, and more studies are needed in the future.

Studies have demonstrated the participation of lncRNAs in the development of the tumor immune microenvironment (TIME) in breast cancer [23]. Here, the risk model established based on ARL also reflected the TIME. Differences in the ICI landscape were observed in breast cancer patients by risk stratification. LncRNAs play a critical role in modulating the TIME and reshaping the immune landscape [24]. During tumor growth, the emergence of fresh blood vessels is triggered by factors like hypoxia-induced TIME induction, enabling the acquisition of necessary blood supply [25]. The crosstalk between abnormal tumor blood vessels and various immune cells determines the immune state in TIME [25]. Consequently, ARLs are worthy of further investigation in terms of regulating the TIME and breast cancer progression. Exploring the potential of lncRNAs could be a hopeful approach for treating breast cancer, given the growing significance of anti-angiogenic therapy.

We also noticed enrichment of ARLs in PI3K-AKT, PPAR, and IL-17 pathways in breast cancer. Mutation of the PIK3CA gene leads to the activation of the PI3K pathway and chemoresistance [26]. Targeting the PI3K-AKT pathway has shown promising preclinical activity in breast cancer [27]. PPAR has a significant impact on cellular differentiation, inflammation, metabolism of glycolipids, regulation of the immune system, and the development of tumors [28, 29]. There have been reports suggesting a strong correlation between PPAR and breast cancer at a biological level, however, the specific involvement of PPAR in breast cancer remains largely unexplored [30–32]. IL-17 also has a direct effect on tumor cells to alter gene profiles, making the cells more aggressive and favoring tumor growth *in vivo* [33]. Furthermore, in breast cancer, breast tumor cells induce γδ T cells to produce IL-17, which enables the formation of the CD8 + T cell-suppressive phenotype and creates an environment conducive to disease progression, in turn, leading to distant metastasis [34].

The newly developed eight-lncRNA signature contained several lncRNAs that have been reported to be related with breast cancer. CYTOR, also known as LINC00152, was upregulated in breast cancer [35] and related with advanced stage, lymphatic invasion, and shorter OS of patients [36]. CYTOR could facilitate breast cancer growth and tamoxifen resistance by targeting KLF5 and miR-125a-5p [37, 38]. Moreover, the lncRNA SNHG10 was revealed to suppress doxorubicin resistance in triple-negative breast cancer [39]. Additionally, MAFG−DT has emerged as a newly identified biomarker that indicates the risk prognosis in breast cancer [40]. In line with our discoveries, the lncRNA OTUD6B−AS1 was found to enhance resistance to paclitaxel in breast cancer [41], as well as being associated with an unfavorable prognosis [42]. Among the eight prognosis-related lncRNAs in this study, OTUD6B−AS1 was selected for further research because it showed the largest HR value in the univariate and multivariate analyses. Our results proved that higher expression of OTUD6B−AS1 was positively related with larger tumor size, positive lymph node metastasis, more advanced tumor stage, and higher Ki-67 expression in breast cancer, indicating poor survival of patients with breast cancer. OTUD6B−AS1 also promoted breast cancer cell proliferation, wound healing, migration, invasion, and HUVEC tube formation, and mechanistically regulated EMT- and angiogenesis-related molecules. However, the remaining lncRNAs identified in our prognostic signature have been poorly investigated. More comprehensive studies on functional relevance and molecular mechanisms remain to be performed in the future. Moreover, multicenter and large-scale studies should be conducted in order to validate the prognostic value of the eight–lncRNA signature.

To sum up, we evaluated eight ARLs as a risk assessment tool for forecasting the prognosis and assessing the impact of immunotherapy in breast cancer. OTUD6B−AS1 potentially facilitated the advancement of breast cancer by regulating molecules associated with EMT and angiogenesis, indicating a potential target for therapeutic intervention in breast cancer.

## MATERIALS AND METHODS

### Transcriptome matrix and clinical data collection

The TCGA database provided the clinical information and transcriptome matrix of breast cancer samples. To be considered eligible for sample screening, the samples needed to have both transcriptome expression data and prognostic information. Exclusion occurred for patients whose survival times were not available. For further analysis, a grand total of 1069 samples of breast cancer were incorporated. The gene matrix of each sample was extracted and merged using Perl scripts.

### Identification of ARLs

The angiogenesis genes were collected from the hallmark gene sets (http://www.gsea-msigdb.org/). Perl scripts and the R package “limma” were used to extract the expression of angiogenesis-related genes. The ARLs were screened through Pearson correlation analysis. With the threshold of |correlation coefficient| > 0.3 and P < 0.001, 464 ARLs were subjected to subsequent analysis (Supplementary Table 1).

### Risk model construction of ARLs

Univariate Cox regression was used to screen the prognostic ARLs using the R package “survival.” The LASSO algorithm was further employed to identify prognostic ARLs for breast cancer using the R package “glmnet”. Utilizing multivariate Cox regression, the prognostic ARLs were selected and a risk model was constructed. The risk score of each sample was computed using the formula: = (–3.605 × AL118556.1 expression) + (–0.726 × SNHG10 expression) + (–0.442 × MAFG−DT expression) + (–0.887 × AC097478.1 expression) + (0.665 × OTUD6B−AS1 expression) + (0.388 × CYTOR expression) + (–0.469 × AL357054.4 expression) + (–1.38 × MED14OS expression). The coefficient in this formula represented the corresponding HR (hazard ratio) value of each lncRNA in the multivariate Cox regression. Kaplan–Meier survival curve was used to estimate the OS, PFI and DSS rate of patients in the low-risk and high-risk groups using the R package “survival”. Principal component analysis (PCA) was utilized to observe the separation pattern of patients in the two groups using the R package “ggplot2”. The breast cancer samples were randomly classified into training cohort and validation cohort at a ratio of 7:3 through the “caret” R script.

### Independence evaluation of the risk model

Cox regression analyses were employed to investigate the independence of the risk model using the R package “survival”. A nomogram was constructed to estimate the 1-, 3- and 5-year survival using the R package “rms”. The prognostic performance of the risk model was validated through time-dependent ROC analysis using the R package “timeROC”.

### Immune cell infiltration landscape

Using the “estimate” R package, we calculated the immune function score of breast cancer samples. The CIBERSORT algorithm was used to investigate the components of the immune infiltration landscape. Using “CIBERSORT R script v1.03”, 22-types immune cells were calculated. An ssGSEA algorithm was utilized to assess the components immune cells via the “GSVA” R package.

### Tumor mutational burden

The TCGA database provided the tumor mutation data of breast cancer samples in “maf” format. The mutation data was obtained from the raw data using Perl scripts, and a waterfall diagram was created using the R software package called “Maftools”.

### Immune response and drug sensitivity

The TCIA database provided the immunophenoscore (IPS) outcome. The TIDE database was used to analyze Tumor Immune Dysfunction and Exclusion (TIDE). Drug sensitivity (IC50) was obtained via the Genomics of Drug Sensitivity in Cancer (GDSC) database, via the R package “pRRophetic”.

### Functional enrichment

The R package “limma” was utilized in order to identify differentially expressed genes (DEGs) (|Fold Change| ≥ 1.4 and *P*-value < 0.05) in the low-risk group and high-risk group. Metascape database was used to enrich the mechanism of DEGs, and KEGG analysis was used to reveal the potential pathways using the “clusterProfiler” R package [43].

### Tissue specimens

Thirty-six breast cancer fresh tissues were collected at Qilu Hospital of Shandong University from February 2022 to November 2022. All patients signed informed consent. This work was approved by the Ethics Committee of Qilu Hospital of Shandong University (KYLL-202111-021-1).

### RNA extraction and quantitative real-time PCR (qRT-PCR)

RNAiso Plus (Takara, Code No 9109) was used to extract RNA in breast cancer tissues and cells. cDNA was synthesized with the Geneseed® II First Strand cDNA Synthesis Kit (Geneseed, GS0201-2). Geneseed® qPCR SYBR® Green Master Mix (Geneseed, GS0201-3) was used for qRT–PCR on an ABI 7500 Real-time PCR system, with GAPDH as a control. The expression of OTUD6B−AS1 was calculated using the 2-ΔΔCt method. The primer sequences are OTUD6B−AS1-F: 5′-AATTGGCTAGAGCGCCAGA-3′, OTUD6B−AS1-R: 5′-GGGGCGGTATTACGACCTTTT-3′; GAPDH-F: 5′-AGAAGGCTGGGGCTCATTTG-3′, GAPDH-R: 5′-GCAGGAGGCATTGCTGATGAT-3′.

### Cell culture and transfection

The BT474 cells (Cell Bank of the Chinese Academy of Sciences, Shanghai, China), were cultured in RPMI-1640 (Gibco, Carlsbad, CA, USA). Human umbilical vein endothelial cells (HUVEC) were grown in Dulbecco’s Modified Eagle’s Medium (Gibco, Waltham, MA, USA). All cells were incubated in 5% CO_2_ incubators at 37° C. The OTUD6B−AS1 overexpression plasmid (pcDNA3.1-OTUD6B−AS1) synthesized by GENERAL BIOL (Anhui, China) was transfected using TurboFect (Thermo Fisher Scientific, Waltham, MA, USA).

### Cell counting kit-8 (CCK-8) assay

Transfected cells were inoculated into 96-well plates at 5,000 cells per well. The wells were then treated with 10 μL CCK-8 reagent (Bestbio, Shanghai, China) at 24, 48, 72, and 96 h and incubated at 37° C for 2 h prior to measurement.

### Wound healing and cell migration and invasion assays

Using a 10-μL pipette tip, a layer of transfected cells on 6-well plates was scratched. Photographs capturing the process of wound healing were taken at both 0 and 24 hours.

Cell migration and invasion were detected using Transwell inserts. The number of cells stained with crystal violet was counted under × 200 magnification.

### Tube formation assay

HUVEC were placed in 96-well plates that had been previously coated with 50 μL of Matrigel. Subsequently, 200 μL of conditioned medium from transfected BT474 cells was added. Tube formations were captured under a 40-fold magnification after a duration of 12 hours.

### Western blot

For the western blot analysis, the antibodies used were as follows: E-cadherin (AF0131, 1:1000, Affinity), VEGFA (sc-57496, 1:200, Santa Cruz), SMAD5 (ab40771, 1:5000, Abcam), MMP1 (A0568, 1:1000, Boster), HIF1α (sc-13515, 1:200, Santa Cruz), Snail (3099-1-AP, 1:1000, Proteintech), and Twist1 (5465-1-AP, 1:1000, Proteintech), with GAPDH (AB0037, 1:5000, Abways) used as an internal control.

### Statistical analysis

Analysis was performed using R software (version 4.1.0), Perl scripts, SPSS 20.0, and GraphPad Prism 5.0. Differences were analyzed using the Wilcoxon rank-sum test between the two groups, or one-way analysis of variance (ANOVA) among three groups. Correlations between OTUD6B−AS1 expression and the clinicopathological features were explored using chi-square test or Fisher’s exact test. P < 0.05 was considered as statistically significant.

### Availability of data and materials

Data and clinical information involved in this paper were obtained from a public database (https://portal.gdc.cancer.gov/). We have provided a detailed GitHub project with the link: https://github.com/MarkPinky/breast-cancer.git. The datasets used and/or analyzed during the current study are available from the corresponding author on reasonable request.

## Supplementary Material

Supplementary Figure 1

Supplementary Table 1
