# Supplementary material for: An angiogenesis-related lncRNA signature predicts the immune microenvironment and prognosis of breast cancer
Source: Aging (Albany NY). 2023 Aug 3;15(15):7616–36. doi: 10.18632/aging.204930 (PMC10457060; doi:10.18632/aging.204930)
Supplement: Supplementary Figure 1 [file aging-15-204930-s001.pdf]

SUPPLEMENTARY FIGURE

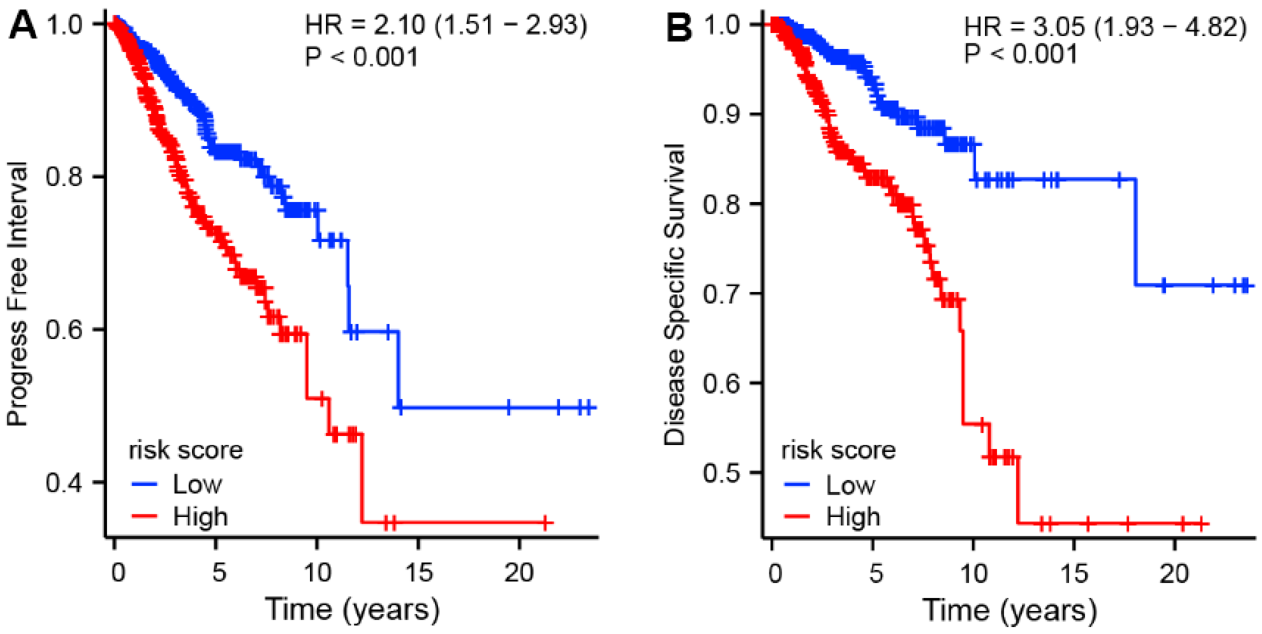

Supplementary Figure 1. Kaplan-Meier survival curve analysis suggested that the Progress Free Interval (PFI, **A**) and Disease Specific Survival (DSS, **B**) time of patients with high-risk score were shorter than those with low-risk score (P < 0.001).
